# A review of epidemiology, clinical features and disease course, transmission dynamics, and neutralization efficacy of SARS-CoV-2 variants

**DOI:** 10.1186/s43168-021-00090-x

**Published:** 2021-11-06

**Authors:** Paroma Deb, Md. Maruf Ahmed Molla, KM Saif-Ur-Rahman, Manik Chandra Das, Debashish Das

**Affiliations:** 1 Department of Virology, Dhaka Medical College, Dhaka, 1217 Bangladesh; 2 Department of Virology, National Institute of Laboratory Medicine and Referral Center, Dhaka, 1207 Bangladesh; 3 Health Systems and Population Studies Division, ICDDRB, Dhaka, Bangladesh; 4 Department of Public Health and Health Systems, Graduate School of Medicine, Nagoya University, Nagoya, Japan; 5 Department of Public Health, University of South Asia, Dhaka, Bangladesh; 6 Institute of Global Health, University of Geneva, Geneva, Switzerland

**Keywords:** SARS-CoV-2, COVID-19, Epidemiology, Immune evasion, Vaccine, Monoclonal antibody, Disease course

## Abstract

**Background:**

After the first detection in November 2019, SARS-CoV-2 has spread rapidly over the continents and started the pandemic of the millennium. In addition to several novels and repurposed monoclonal antibodies (mAbs) as a therapeutic option against COVID-19, scientists from across the world have developed several candidate vaccines, developed mainly targeting the Wuhan strain, with very promising results to combat this pandemic. Unfortunately like any RNA viruses, SARS CoV-2 has also gone through the accumulation of hundreds and thousands of mutations in their genome lead to the development of several variants of concerns (VOC) and variants of interests (VOI), resulting in increased transmissibility and virulence of the virus, along with their capacity to escape cross-protection. Seemingly, the main hindrance of containing this pandemic right now is the effectiveness of currently available vaccines and mAbs against newly emerging variants. Therefore, it is important to monitor variants epidemiology, transmission dynamics, clinical characteristics, as well as their immune evasion capacity to implement appropriate vaccine strategy and other containment measures.

**Body:**

In this review, we tried to focus on variants characteristics and to what extent they can escape immunity, provided by both available vaccinated sera and convalescent sera. A stringent literature review was performed using various databases, mentioned in the methodology portion. The current geographical distribution of these variants of SARS CoV-2 has been presented using a heat map. Findings from published articles comparing these variants, in terms of genome epidemiology, transmissibility, viral load dynamics, and association with different waves have been described briefly. Due strength was given while describing variants neutralization potency against current vaccines, mAbs, and also against convalescent sera. Data from both clinical trials and in vitro/ex-vivo studies have been discussed here. Comparative findings from several articles were brought into one concise paper. After careful reviewing of all the available data, it was clear that, without hesitation, we should strengthen our vaccination strategy, because the severity of COVID 19 is reasonably lower, irrespective of variants and vaccine used.

**Conclusion:**

We hope that many falsified myths and beliefs regarding vaccine immunity and emerging variants will be clarified in light of this available evidence, which we summarized in our paper.

## Background

The first cases of SARS-CoV-2 infection were detected near a seafood market in Wuhan, China [1]. On March 11, 2020, the World Health Organization (WHO) declared the disease as a global pandemic [2]. To date, more than 164 million cases have been detected throughout the world with the death toll amounting to more than 3.4 million [3].

With the time being passed, SARS-CoV-2 has gone through several genetic evolutions with no significant impact on cross-immunity, until the recently emerged strains, namely, Alpha variant (previously known as UK variant) (B.1.1.7), Beta variant (previously known as South African variant) (B.1.351), Gamma variant (previously known as Brazilian variant) (P.1), Delta variant (previously known as Indian variant) (B.1.617), and other variants of interest (VOI) [4, 5]. They are raising concerns as many of their mutations are positioned in the S1 portion of SARS-CoV-2 spike protein, against which the majority of candidate vaccines and monoclonal antibodies (mAb) are directed, and thus they can affect antibody-mediated immunity [5–7]. Furthermore, there are concerns that these variants may have significantly different clinical course or transmission dynamics compared to wild-type viruses [8, 9].

According to the latest published data, more than 100 candidate vaccines have been going through different phases of clinical trials with another 184 in pre-clinical phases of development [10]. Among them, five vaccines (BNT162b2 by Pfizer-BioNTech; mRNA-1273 by Moderna; ChAdOx1 nCoV-19 (AZD1222) by Oxford-AstraZeneca; Ad26.COV2-S by Janssen; and lastly Sinopharm BBIBP-CorV vaccine developed by Sinopharm, China) have received emergency use authorization (EUA) [11–15]. Several monoclonal antibodies (mAbs) have been developed and some of them have received EUA to treat COVID-19 cases [6, 16, 17]. To understand the extent these new variants are influencing the efficacy of currently available vaccines or mAbs, and to formulate an effective prevention and treatment strategy against the virus, a careful monitoring of variant-associated mutations, symptoms, clinical course, transmission dynamics, and other variables should be documented. Hence, in this review, we comprehensively summarize all the relevant literature documenting SARS-CoV-2 variants including their epidemiology, clinical symptoms, disease course, transmission dynamics, and neutralization by vaccines, convalescent sera, and mAbs.

## Main text

### Methodology

Publicly available databases and COVID-19 information repositories such as PubMed including Medline, Google Scholar, WHO, GISAID, CDC, Worldometers, Our World in Data, and several other sources were used to search for suitable articles. Peer-reviewed publications, as well as epidemiological updates and news pieces, from December 2019 to August 2021 were evaluated and if deemed suitable, authors downloaded full text. Search terms include, but are not limited to, SARS-CoV-2, COVID-19, symptoms, disease severity, variants, monoclonal antibody, vaccine, convalescent sera, epidemiology, B.1.1.7 (alpha), B.1.351 (beta), P.1 (gamma), B.1.617.2 (delta), and different combinations of listed search terms. Information was stored in Microsoft Excel and Word. A geographical heat map of COVID-19 variants was created using the Plotly in Python.

### COVID-19 variants in circulation

SARS-CoV-2, a 29.9 Kb positive-sense single-stranded RNA virus, much like its predecessors and other viruses in the coronavirus family, such as MERS-CoV and SARS-CoV, mutates constantly, although the mutation rate is considerably slower than many other RNA viruses [18]. The more people the virus infects, the probability of acquiring genetic changes increases. Luckily, most of the genetic changes the virus acquires have little impact on the ability of the virus to cause severe disease or increased infection. But, at times, the virus accumulates genetic changes that confer competitive advantage and help the virus to transmit more easily or cause severe disease in infected individuals, through alteration of major proteins, such as that of spike protein in SARS-CoV-2 tasked with binding to a receptor through the receptor-binding domain (RBD) (spike protein residue 319-541) and entry of virus within host cells [18–20].

**Fig. 1:**
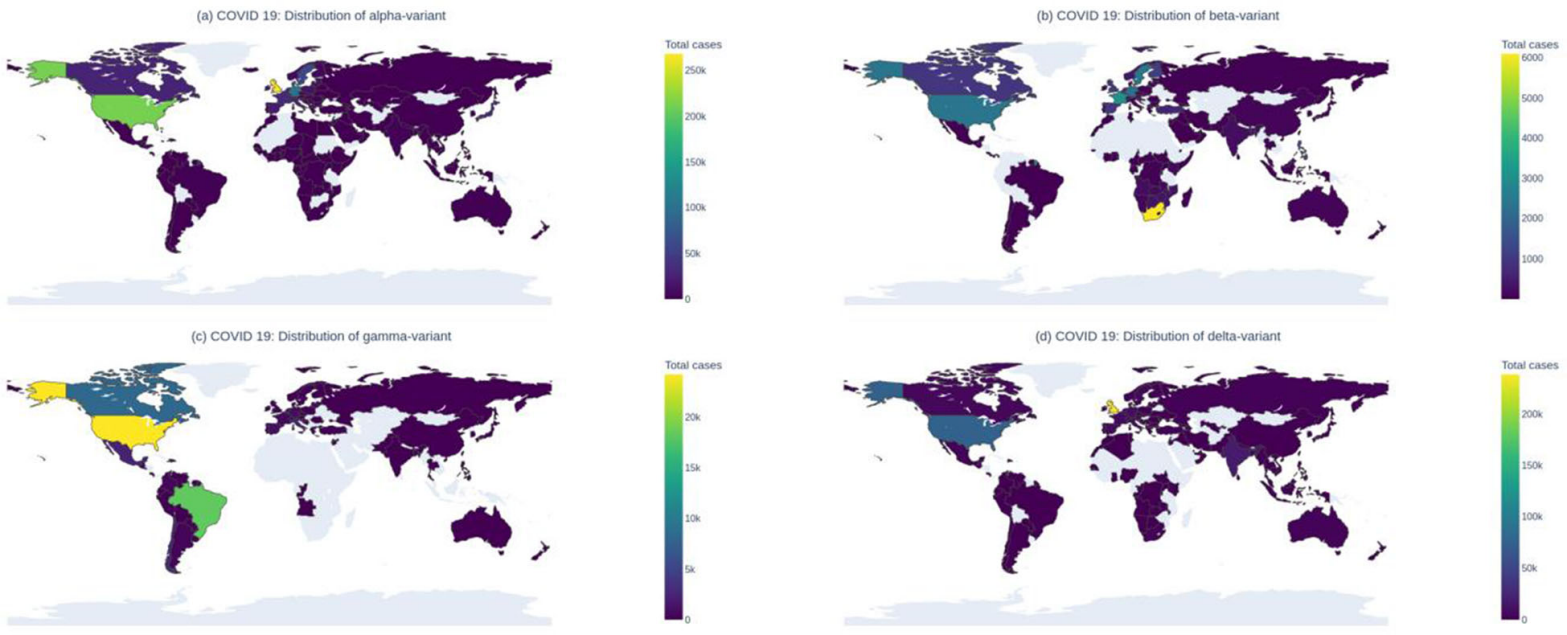
Geographical distribution of major COVID-19 variants (**a**—Alpha, **b**—Beta, **c**—Gamma, and **d**—Delta) [22]

The original strain of SARS-CoV-2 that originated in Wuhan, China toward the end of 2019, mutated, albeit at a slower pace, and gave rise to other variants, defined as strains with significant phenotypic changes to the original strain [19], which now are predominant strains in different countries around the globe including UK, USA, India, South Africa, Brazil, and several other nations (Table 1). Some of these variants are described as variants of concern (VOCs), as classified by WHO, due to their ability to evade the host immune system, increased transmissibility, decreased efficacy of vaccination and therapeutic interventions, and the potential increase in disease severity. In addition to VOCs, several other variants of interest (VOI) are under observation with no variant of high consequence identified thus far [4, 5, 21]. In addition to VOC and VOI, several variants, with no clear-cut evidence on increased transmissibility or virulence, are under monitoring including P.2, P.3, R.1, R.2, B.1.466.2, B.1.621, B.1.427/B.1.429 (formerly known as epsilon and denoted as VOC in North America) etc. [21]. A geographical heatmap of major COVID-19 variants is provided in Fig. 1.

**Table 1:** Major SARS-CoV-2 Variants in circulation

WHO label	Pango Lineage (VOC/VOI/VUI)	GISAID Clade	Originating country	First detectionL452R, E484Q, P681R	Significant mutations
Alpha	B.1.1.7 (VOC)	GRY	Kent, UK	In November 2020 (Sample from September 2020)	N501Y (mutation in RBD), 69-70del (Spontaneous Spike mutation), P681H (mutation near S1/S2 furin cleavage site),
Beta	B.1.351 B.1.351.2 B.1.351.3 (VOC)	GH/501Y.V2	South Africa	Nelson Mandela Bay, South Africa in May 2020	N501Y, K417N, E484K (Mutation in RBD of spike protein)
Gamma	P.1 P.1.1 P.1.2 (VOC)	GR/501Y.V3	Brazil	Brazil in November 2020	N501Y, K417T, E484K (Spike protein mutations)
Delta	B.1.617.2 AY.1 AY.2 AY.3 (VOC)	G/478K.V1	India	Maharashtra, India on 5 October 2020	E484Q, L452R, P681R (Spike protein mutations)
Eta	B.1.525 (VOI)	G/484K.V3	Nigeria, UK	December 2020 in UK and Nigeria in December 2020	E484K, F888L (Mutation in S2 domain of spike protein), 69-70del (Spontaneous Spike mutation),
Iota	B.1.526 (VOI)	GH/253G.V1	USA	New York, the USA in November 2020	T95I, E484K, D253G, D614G (Spike protein mutations)
Kappa	B.1.617.1	G/452R.V3	India	October 2020	L452R, E484Q, P681R
Lambda	C.37 (VOI)	GR/452Q.V1	Peru	December 2020	G75V, T76I, Δ246-252, L452Q, F490S, D614G, T859N

### Clinical features, transmission, and disease course:

#### Classical clinical features

Earlier literature reporting COVID-19 disease in the Chinese population reported fever, dry cough, breathing difficulties, headache, and pneumonia as the most common presenting complaints [23]. However, most of the early cases had contacts with the Wuhan seafood market and hence, once the virus spread to other countries, diverse symptoms started to emerge and were reported in scientific literature. A recent meta-analysis of 152 publications from 23 countries including a total of 41409 participants demonstrated 26 symptoms experienced by COVID-19 infected patients. Among them, fever was the most frequently experienced symptom (58.66%) followed by cough (54.52%), dyspnea (30.82%), malaise (29.75%), fatigue (28.16%), and sputum secretion (25.33%) [24]. Other reported symptoms include neurological symptoms including loss of taste and smell, dermatological manifestations, myalgia, sneezing, sore throat, rhinitis, diarrhea, anorexia, chest pain, and headache. But in studies with participants more than 100, fever, cough, and dyspnea were the three most reported symptoms.

#### Infection with variants

The Office for National Statistics (ONS) in the UK surveyed people testing strong positive for COVID-19 in polymerase chain reaction (PCR) test and compare the symptoms with that of the original strain. The survey data did not reveal any unusual symptoms with the classic reported symptoms making up the bulk of the cases (Table 2) [25]. This was further reconfirmed in a recently published study in the UK that revealed patients affected with alpha reported similar symptoms to that of other widely circulating strains and there are no discernible differences between alpha variant and other strains [26]. Regarding the widely circulating delta variant, symptoms might differ from other circulating strains, as suggested by the Zoe COVID study in the UK. People infected with delta variant, both vaccinated and unvaccinated, primarily report symptoms such as runny nose, headache and sneezing, and sore throat [27]. While traditional symptoms such as anosmia and fever are still reported, the percentages are small compared to previous months.

**Table 2:** Comparison of symptoms between alpha variant and wild strain infected individuals in the UK

Symptom	Variant	Original strain
Cough	35%	28%
Fatigue/Weakness	32%	29%
Headache	32%	30%
Muscle aches	25%	21%
Sore throat	22%	19%
Fever	22%	20%
Loss of taste	16%	19%
Loss of smell	15%	19%

Similar comments were made by other researchers around the globe regarding beta and gamma variants and other variants of interest (VOI), as symptoms experienced by affected individuals were similar to that one of wild SARS-COV-2 strains widely prevalent during the early months of the pandemic.

### Transmission dynamics

#### D614G mutation—wild strain

While SARS-CoV-2 sequences deposited in global repositories in February 2020 did not reveal this mutation to any of the sequences, the number of sequences with D614G mutation increased rapidly in the next months, reaching 70% in May, suggesting a transmission advantage over D614 from the original Wuhan strain [28]. It is postulated that the spike protein of SARS-CoV-2 is largely responsible for viral tropism and increased transmissibility. In previous researches, it was found out that sequences bearing D614G mutation were linked to higher viral loads in the upper respiratory tract [29, 30]. While the exact mechanism for that is yet to be understood but initial research suggests that D614G enhances viral cell entry as the G614 S-protein trimer being more suitable for ACE2 binding [29, 30].

#### Alpha variant

By analyzing the COG-UK datasets, researchers found out that the reproduction number (*R)* for the alpha variant is 43–90% more than other prevailing variants in the UK [31]. This finding was similar when analyzing data from other countries such as Denmark, Switzerland, and the USA [31]. Several hypotheses have already been proposed by researchers regarding increased transmissibility of alpha variant including a longer infectious period, ∆H69/∆V70 deletion-mediated immune escape, and high viral load as observed through low CT-values obtained during PCR testing [32–34].

#### Beta variant

Compared to other strains, infection with the beta variant leads to increased viral load, much like the alpha variant, resulting in increased human-to-human transmission. This has been evidenced by the recent surge in beta variant cases in South Africa where, at the start of December 2020, close to 90% of new cases were infected by this variant. But at the start of October 2020, only 11% of case sequences were of this variant [8]. Similarly, in Bangladesh, the second wave was initiated with the rapid spread of beta variant, reaching up to 93% of total sequenced cases and increasing reproduction number (reaching up to 2 in March 2021 compared to 1 throughout 2020) [35, 36]. Another country, Zambia, did not report any cases of beta variant from March 2020 to early December 2020. But in mid-December, 22 out of 23 cases were detected as beta variant and this correlates with a 16-fold increase in infection within the country within a span of 1 month starting from December 2020 [8].

#### Gamma variant

Regarding gamma variants currently prevalent in Brazil, research results, based on data from Manaus, estimate that gamma variant cases are 1.7-2.4 times more transmissible than non-P.1 lineage despite higher seroprevalence among residents in Manaus compared to neighboring regions [37]. Another study conducted using publicly available data on hospitalized patients found the transmissibility of gamma variant, after adjusting for other variables, to be 2.5 times more than non-gamma variant [38].

#### Delta variant

The latest VOC declared by the WHO, the delta variant, currently spreading rapidly in India and neighboring Nepal and Sri Lanka, Bangladesh, and 100 other countries including UK and USA, is thought to have greater transmissibility than other lineages, prevalent in both UK and India, largely due to a double mutation in spike protein, E484Q, and L452R, that are responsible for higher viral load, increased transmissibility, and immune escape [39]. Recent data suggest that viral load in delta variant-infected individuals is 1000 times greater than people infected with the original strain. The variant is twice as much transmissible as the original strain [40].

### Disease course

#### Alpha variant

A community-based study including a large British dataset, that depended on absence/presence of S-gene amplification in real-time PCR to determine B.1.1.7 lineage, of 2.2 million COVID-19 affected people from September 2020 to February 2021 revealed a 61% (CI: 42–82%) increased mortality among population infected with the alpha variant overpopulation infected with other strains [41]. This was further reconfirmed by The Office for National Statistics as patients with S-gene negative status were found to be appearing for a test earlier than the other group, suggesting a more rapid and severe disease course [25, 42]. Similar findings were obtained from a study conducted using data, comprising of 19995 cases, from seven European countries. Increased rate of hospitalization was observed in VOC cases (B.1.1.7; P.1; B.1.351) compared to non-VOC cases [43]. Furthermore, on unmatched analysis, B.1.1.7, B.1.351, and P.1 cases were 2.3, 3.3, and 2.2 times more likely to get admitted into ICU [43]. Other studies conducted by Challen et al. and Grint et al. revealed comparable findings with mortality hazard ratio after 28 days being two-thirds higher in VOC groups (1.64 and 1.67 respectively) compared to non-VOC cases [44, 45]. But these findings were challenged by a group of researchers who sequenced 341 samples and compared between sequences of B.1.1.7 lineages and non-B.1.1.7 lineages. While there is evidence that viral load and disease transmission is higher in B.1.1.7 lineages, no association could be established between severe disease and infection with alpha variant [34].

#### Beta variant

While no data regarding increased disease severity in individuals infected with the beta variant could be found as of writing this paper, in several countries such as South Africa, Zambia, and Asian countries such as Bangladesh, this variant has outcompeted other circulating variants and has led to increased transmission of the virus, and as a consequence, resulting in higher morbidity and mortality [8, 35]. Earlier reports suggest in admission mortality to be 20% higher in South Africa during the second wave of pandemic compared to the first wave. Again, no definitive answer could be provided regarding the association of the beta variant with increased mortality, as it could be down to a higher number of cases and strain on the healthcare system. In Bangladesh, despite an increase in case numbers, interestingly, the case fatality rate (CFR) has decreased compared to earlier months when others strains were prevalent within the country [46].

#### Gamma variant

While there is little research evidence on the increased mortality and morbidity caused by the gamma variant, recent evidence suggests that the variant may be responsible for increased case fatality rate (CFR) in all age groups with CFR increasing significantly in February, compared to data from January among residents in Parana state in Brazil [38]. Similar findings were observed among residents in Manaus where the mortality is estimated to rise 1.2 to 1.9 times since the emergence of gamma variant compared to previous periods [37]. But it remains to be elucidated whether the apparent rise in mortality is related to gamma variant rather than increased transmissibility and dwindling healthcare resources in Brazilian hospitals.

#### Delta variant

Actual data emanating from India reveal that, while there is a catastrophic rise in case of numbers in March and April with 0.4 million cases being detected each day, CFR has decreased and remained low throughout the duration [47, 48]. In a recent study conducted in Ontario Canada, delta variant-infected people were 108% more likely to get hospitalized compared to other strains. Regarding ICU admission and death, there was a 234% and 132% increase in risk compared to other strains [49]. Similarly, studies conducted in Scotland and Singapore revealed that people infected with the delta variant are more likely to get hospitalized, have higher odds of oxygen requirement, and suffer for a longer duration compared to other strains [50, 51].

### Neutralization potency of post vaccinated sera

#### Alpha variant

Reduction of neutralization against full panel spike mutation (UKΔ8) of B.1.1.7 (alpha variant) pseudovirus and pseudoviruses carrying either single (N501Y) and triple mutations (N501Y, A570D, and the 69/70 deletion) have been tested with serum, vaccinated with BNT162b2 vaccine, mRNA-1273, and ChAdOx1 vaccine in several in vitro settings. For selected insertion, either single or triple, reduction in titers varied from no reduction to highest 3.3-fold decrease, whereas UKΔ8 resulted in highest fold change of 5.83 in 80–85 age group and lowest fold change of 1.78 in 60–65 age groups in cases of BNT162b2 vaccine, 1.8-fold for mRNA-1273 vaccine, and 9 times reduction in neutralization titer ChAdOx1 vaccine. Serum was tested against USA/WA-1/2020 background strain, wild-type SARS-CoV-2, or against Victoria virus strain and was taken after 2^nd^ dose of vaccination. Despite this reduction in GMT, it is unlikely for the UK variant to escape vaccine-mediated protection because Smith et al. previously for influenza virus vaccine sufficed that, more than 20% reduction in neutralization titer was needed for vaccine immune evasion by newly emerging virus strains [52–60].

As for the clinical efficacy, the BNT162b2 vaccine showed effectiveness ranging from highest 95.3% in Israel and lowest 87% in Qatar against this VOC, assuming 94.5% and 44.5% prevalence rate of B.1.1.7 in those countries at the time of clinical trials. Most importantly, two doses of the BNT16b2 vaccine were capable of preventing hospitalization as a whole in 97.5% of cases and critical hospitalization in 96.7% of cases [61, 62]. As for the ChAdOx1 vaccine, in a clinical trial after receiving two doses of ChAdOx1 vaccine, they observed that the clinical efficacy against symptomatic COVID-19 is 70.4% [56].

#### Beta and gamma variant

These two variants, recently named as beta and gamma variants, share identical triplet mutations (E484K, K417N/T, and N501Y) in RBD and thus presumed to show the same extent of immune evasion capabilities, but surprisingly B.1.351 showed more resistance to antibody neutralization than P.1. BNT162b2 vaccinated sera showed a 2.6-fold and 6.7-fold reduction in GMT in two different studies, while ChAdOx1 and mRNA-1273 vaccinated sera showed 2.9- and 4.5-fold reduction in GMT against P.1 variant, compared to Victoria virus strain **[**60, 64]. When overall B.1.351 variant was considered, reduction in vitro study titer varied from 6.5- to 7.6-fold for sera vaccinated with BNT162b2 vaccine and 9-fold and 8.6-fold for sera vaccinated with ChAdOx1 and mRNA-1273 vaccines. Although, when all three strains (v1, v2, v3) of B.1.351 lineage were investigated separately, the highest reduction of titer was observed for v3 with a reduction of 42.4-fold for BNT162b2 and 19.2-fold for mRNA-1273 followed by v2 with 41.2-fold for BNT162b2 and 20.8-fold for mRNA-1273 and lastly of v1 with 34.5-fold for BNT162b2 and 27.7-fold for mRNA-1273 vaccine sera [55, 59, 63, 64]. Interestingly, Tada et al. and Wu et al. showed that, whether it is E484K, K417N-E484K-N501Y triple, or full panels of B.1.351 mutation, neutralization capacities of post vaccinated sere were reduced but not completely abolished, rather, were able to provide cross-protection with sufficiently high titer in in-vitro settings [58, 59, 65].

Fortunately, real-life scenarios for these in vitro studies have shown more promising results, especially in terms of preventing severe diseases. Effectiveness of BNT162b2 vaccine against beta variant among the residents of long-term care facilities in France, after two complete doses of vaccination, was 49% but against severe disease VE remained high with 86% [66], while, in Qatar, in the background of 50% beta variant prevalence, VE ranged from 72.1 to 75% based on cohort and case-control study design [61]. Similarly, AstraZeneca-Vaxzevria was found protective against infection, hospitalization, and death, among ≥ 60 years, in a setting of the high prevalence of gamma variant in Brazil, in 77.9%, 87.6%, and 93.6% vaccinated individuals respectively [67].

#### Delta variant

The most recent VOC, responsible for the 3^rd^ wave of infection worldwide, the B.1.617.2 or delta variant has shown its potentials to increase transmissibility as well as enhanced capability of immune evasion. While numerous studies are on track to reveal the level of danger, Edara et al. compared neutralizing antibody response between B.1.617.1 and WA1/2020 variant, in sera vaccinated with mRNA-1273 and BNT162b2 vaccines. Differences in GMT were observed 1142 and 1012, for mRNA-1273 and BNT162b2 mRNA vaccinated sera respectively, against control strain. Despite 6.8-fold less susceptibility, all of the posts vaccinated sera were able to neutralize this VOC, which suggests some level of protective immunity against B.1.617.1 by existing mRNA vaccines [68], while Covaxin immune sera showed probable immune escape capacity with a 1.84 GMT ratio for B.1.617, but 1.06 for B.1.1.7 and D614G variants [69]. On the other hand, Ferreira et al. devoured to identify the most notorious mutation in this variant, and following other studies, they showed that E484K mutation conferred 10-fold reduction and E484Q conferred slightly milder yet significant reduction in neutralization by BNT162b2 vaccinated sera [70].

Some very recent preprint articles concluded that both BNT162b2 and Chadox1nCov vaccines, after two complete doses, are highly effective by conferring 88% and 67% protection respectively against symptomatic disease from B.1.617.2 variant [71, 72], with a single dose of ChAdOx1-19 vaccine is effective for preventing infection in 46.2% but in 79.2% for preventing moderate-severe Covid-19 [73].

A table has been provided with the article, representing the summarized results from the studies that have been exploring (both in-vitro/ex-vivo and clinical efficacy studies) for the impact of current VOCs against BNT162b2, mRNA-1273, Chadox1nCov, and Covaxin immune sera (Table 3).

**Table 3:** Overview of immune evasion capacities for current VOCs against BNT162b2, mRNA-1273, Chadox1nCov, and Covaxin immune sera

VOC	Vaccine name	Neutralization capacity against wild-type/D614G strain/Victoria virus	Assays used	References	Original Trial		References
B.1.1.7	BNT162b2	Single mutation- (=) Full spike mutation- (↓) to (↓↓) CE: Against infection: 87.0% to 95.3%; Against symptomatic infection: 97%; Against hospitalization: 97.5%	VSV based pseudovirus assay	[52, 54, 55]	Pfizer	1. Multinational, placebo-controlled, observer-blinded, efficacy trial 2. 43, 548 participants with 21, 720 vaccine recipients 3. Only 8 cases of COVID-19 among vaccine recipients after 7 days of 2^nd^ dose 4. Vaccine efficacy 95%	[77]
	mRNA-1273	Full spike mutation: (↓)	VSV-based pseudovirus assay	[55]			
	Chadox1nCov	B.1.1.7: (↓↓↓) CE: Against symptomatic infection: 70.4%	Live virus microneutralization assay	[56]			
B.1.351	BNT162b2	Triple mutation: (↓↓↓) Full spike variant: (↓↓) E484K mutation: (↓↓) B.1.351 lineage: v1: (↓↓↓); v2: (↓↓↓); v3: (↓↓↓) B.1.351: (↓↓) CE: Against infection: 72.1% to 75.0%; Against severe disease: 86%	In vitro FRENT assay	[55, 61, 63, 64, 66]			
					Moderna	1. Randomized, observer-blinded, placebo-controlled 2. Study site: USA 3. 30, 420 volunteers with 15,210 in vaccine recipient group 4. Only 11 cases of COVID 19 in vaccine recipient group 5. Vaccine efficacy: 94.1%	[78]
	mRNA-1273	For B.1.351 lineage: v1: (↓↓↓); v2: (↓↓↓); v3: (↓↓↓) For B.1.351: (↓↓↓)	In vitro FRENT assay	[55, 63]			
	Chadox1nCov	Triple mutation: (↓↓↓) B.1.351: (↓↓↓) Triple mutants: drop-in neutralization titer to 85 B.1.351: drop in neutralization titer to 74 In live virus, assay titer ranged from (= to ↓↓↓) CE: Against infection: 10.4%, with no severe cases	In vitro FRENT assay; Pseudovirus assay; Live virus neutralization assay; clinical trial	[79]			
					Oxford-Astrazeneca	1. Blinded, randomized, controlled trial across the UK, Brazil, South Africa 2. Two standard-dose vaccine efficacy 62.1% 3. low dose/ standard-dose vaccine efficacy 90.4% 4. Overall vaccine efficacy 70.4% 5. Dosing interval 21 days	[80]
P.1	BNT162b2	Triple mutation: (↓) P.2 with E484K: (↓↓) P.1 with triple mutants: (↓↓)	In vitro FRENT assay	[63]			
	mRNA-1273	P.2 with E484K: (↓) P.1 with triple mutants: (↓↓)	In vitro FRENT assay	[63]			
	Chadox1nCov	Triple mutation: (↓) CE: Against infection: 77.9%; against hospitalization: 87.6%; against death: 93.6%	In vitro FRENT assay	[63, 67]			
B.1.671	BNT162b2	GMT 164 (↓↓) E484K: (↓↓↓) CE: Against symptomatic infection: 88%	FRNT_50_ assay	[68, 70, 71]			
	mRNA-1273	GMT 190 (↓↓)	FRNT_50_ assay	[68]			
	Chadox1nCov	CE: Against symptomatic infection: 60%; Against moderate to severe disease: 79.2%		[71, 73]			
	Covaxin	GMT ratio for B.1.617: 1.84, while for B.1.1.7 and D614G: 1.06	Pseudovirus assay	[69]			

*VOC* variants of concern; = and arrows indicate fold-reductions in neutralizing activity compared to control strain. =: no reduction; ↓: 1–3-fold reduction; ↓↓: 3–7-fold reduction; ↓↓↓: > 7-fold reduction; *CE* clinical efficacy

### Other vaccines

Surprisingly, newly trialed vaccines from Johnson & Johnson and Novavax showed more promising results against these variants of concerns. Results from a phase III trial with 43,783 participants, conducted by Johnson & Johnson, across the US, Central, and South America and South Africa, showed only 468 symptomatic cases of COVID-19. Although, the full picture of this trial, including percentage of SARS-CoV-2 variants among the infected population or how many cases are among the vaccine recipients, etc. is yet to reveal. In contrast, vaccine trials from Novavax are published in a more detailed form. They showed vaccine efficacy after the post hoc analysis, against B.1.1.7 is 85.6% and against B.1.351 is 60% after two complete doses of vaccine. Further statistical analysis showed that overall vaccine efficacy in HIV-negative participants was 60.1% for the prevention of mild to moderate COVID with a reduction in efficacy against B.1.351 variant with 51% [74–76] (Table 3).

### Neutralization potency of convalescent sera

Convalescent sera, being the last resort of treatment in some settings, for severe COVID, aggravated the need to evaluate its neutralization potential against several variants. More or less similar findings were observed for all of these studies, where they showed no single mutation is capable enough to cause immune escape except E484K, which is thought to be the major attribute for the reduction in antibody neutralization. Against single point mutation (Δ69-70) and N501Y, neutralization efficiency was the same as D614G, although K417N, E484K, and N501Y substitutions were observed 5-fold to 6.8-fold reduction in neutralization titer [54, 58, 81–83]. Likewise, a full mutation panel of B.1.351 showed a discernible negative impact on neutralization capacity of convalescent serum samples with 13.3-fold, 9.4-fold, and 11- to 33.1-fold reduction in titer in comparison to wild-type, D614G, or Victoria strain of SARS CoV-2. As for P.1 strain, a 3.1-fold reduction in GMT was observed when compared to Victoria [55, 63, 64]. Increased resistance of neutralization is also associated with an increase in frequency, as Wibmer et al. showed 70% serum samples with < 100 ID50 against B.1.351 lineage and in nearly half (48%) samples with no detectable neutralization activity, while 63% sample with < 100 ID50 for triple mutant containing K417N, E484K, and N501Y substitutions with only 27% with no detectable neutralization activity [82]. Interestingly, all of these studies contained some number of convalescent serum samples showing neutralizing activity against B.1.351 as efficient as the original strain, and one study showed six individuals with prior COVID 19 or with exposure history and with the highest antibody titer, exhibited significant cross-neutralization for the B.1.351 [59, 63]. A brief overview of all the studies have been summarized and given in a tabulated form in Table 4.

**Table 4:** Overview of immune evasion capacities for current VOCs against convalescent sera

VOC	Neutralization capacity against wild-type/D614G strain/Victoria virus	Assay used	Reference
B.1.1.7 (Alpha)	2.9-fold (↓)	FRNT50 assay	[60]
B.1.351 (Beta)	Triple mutation: 6.8-fold (↓↓) and 5-fold (↓↓) reduction B.1.351: 13.3-fold (↓↓↓), 9.4-fold (↓↓↓), 11 to 33-fold (↓↓↓)	FRENT assay, Pseudovirus assay	[55, 63, 64, 81, 83]
P.1 (Gamma)	3.1-fold (↓↓)	FRENT assay	[63]
B.1.617.2 (Delta)	4-6 fold reduction in neutralization capacity compared to alpha and D614G variant (↓↓)	FRNT_50_ assay	[84]

*VOC* variants of concern; = and arrows indicate fold-reductions in neutralizing activity compared to control strain. =: no reduction; ↓: 1–3-fold reduction; ↓↓: 3–7-fold reduction; ↓↓↓: > 7-fold reduction

### Neutralization potency of monoclonal antibodies

Structural analysis with two monoclonal antibodies, REGN10933 and P2B-2F6, revealed that E484Q and L452R mutation may interrupt the binding of those two monoclonal antibodies with spike protein. The spike protein mutation L452R, found in both B.1.427/B.1.429 and B.1.617 lineages, is thought to be responsible for decreased neutralization efficacy [39, 69, 85, 86]. Regarding alpha and beta variants, 12 mAbs were directed against RBD and six against NTD of spike protein in Vero E6 cells and compared with wild-type strains. Results revealed a marked reduction in neutralization capacities of 910-302 and S30 mAbs against B.1.1.7 [87]. But results were much worse when tested against beta variant as 910-30, 2-15, LY-CoV555 (bamlanivimab), C121, and REGN10933 (casirivimab) showed markedly reduced capacity in virus neutralization, mediated by E484K and K417N mutations [87].

In addition, researchers evaluated the neutralizing efficacy of mAb therapies that are in clinical use. LY-CoV555 alone or in combination with CB6 failed to neutralize beta variant while some sort of activities was retained, though markedly reduced when tested against REGN10933+REGN10987 and COV2-2196+COV2-2130 combinations. In another study, COV2-2196+COV2-2130 retained their activity against all virus variants though there was a 4-fold reduction in neutralization efficacy [88]. But no significant alteration was observed when treated against S309 and Brii-196+Brii-198 combinations [88]. Interestingly, another VOC, the gamma variant, harbors three similar mutations in RBD residues to that of beta variant, namely E484K, K417T, and N501Y, and hence findings from B.1.351 testing should be relevant to P.1 variant also [88].

These findings including the role of E484K and K417N substitutions in mAb neutralization was pronounced in another study. K417N was responsible for a 27-fold reduction in virus neutralization by mAb COVOX-40 in in-vitro assay while no significant change was observed regarding other mAbs tested. But E484K mutation negatively impacted the efficacy of several mAbs including COV2-2196, COV2-3025, COV2-2381, and S2E12, where 4- to 5-fold reductions in efficacy were observed [88]. N501Y mutation observed in all three VOCs was responsible for a slight reduction in neutralization by mAbs and is consistent across studies in different settings [89]. Two other significant mutations P681H and 69-70del did not have a significant effect on mAb efficacy directed toward receptor binding motif (RBM) of RBD [39, 88] (Table 5).

**Table 5:** Neutralization efficacy of EUA approved mAbs against SARS-CoV-2 variants of concern

Monoclonal antibody	Mechanism of action	Alpha-variant	Beta-variant	Gamma-variant	Delta variant
Bamlanivimab (LY-CoV555)	Binds to overlapping sites at RBD and blocks attachment to ACE2 receptors	Retains activity [55, 88, 90]	Complete loss of activity [55, 84, 88, 90]	Complete loss of activity [91]	Complete loss of activity [84]
Etesevimab (LYCoV016/CB6)		Retains activity [55, 88]	High reduction in activity (in combination with LY-CoV555) [55, 88]	High reduction in activity [55, 84, 88, 91]	Retains activity [84]
Casirivimab (REGN10933)		Retains activity [55, 84, 88]	High reduction in activity [55, 84, 88, 91]	High reduction in activity [55, 84, 88, 90]	
	Binds to RBD and blocks attachment of the virus				Retains activity [84]
Imdevimab (REGN10987)		Retains activity [55, 84, 88, 91]	Retains activity [55, 84, 88, 91]	Retains activity [55, 84, 88, 91]	Retains activity/High reduction inactivity [84, 91]
Sotorivimab (VIR-7831, GSK4182136)	Prevents membrane fusion after viral binding	Retains activity [92]	Retains activity [92]	Retains activity [92]	No data available [88]
Tocillizumab	Inhibits binding of IL6 with its receptor IL-6R	No data available	No data available	No data available	No data available

## Conclusions

A major limitation of these studies is that almost all of them are performed using engineered pseudoviruses and without any serological correlations. Therefore, the role of humoral and cell-mediated immunity is mainly assumption-based. Secondly, complement-mediated cell lysis and antibody-dependent cell-mediated cytotoxicity or phagocytosis are overlooked, which can play either a protective or harmful role in-vivo situations. For convalescent serum samples, the timing of sample collection may not be optimal. If samples were collected early, titer may rise further or wane with time, thus, study results may become compromised. Another important point is there were some discrepancies among the results from pseudovirus and live virus neutralization assay, which should be resolved only with clinical data against all of the variant viruses. At last, laboratory-based results are only half-truth as they do not represent a complex mechanism of the human body. Therefore, more clinical trials are the demand of time to visualize the impact of emerging viruses on cross-protection either by vaccination or by the previous infection.

## Data Availability

Available from the corresponding author on request.

## Abbreviations

SARS CoV-2: Severe acute respiratory syndrome coronavirus-2
COVID-19: Coronavirus disease-19
SARS CoV: Severe acute respiratory syndrome coronavirus
MERS CoV: Middle East respiratory syndrome coronavirus
VOC: Variants of concern
VOI: Variants of interest
RBD: Receptor binding domain
RBM: Receptor binding motif
mAb: Monoclonal antibody
WHO: World Health Organization
GISAID: Global initiative on sharing all Influenza data
CDC: Communicable disease control
EUA: Emergency use authorization
ONS: Office for National Statistics
PCR: Polymerase chain reaction
NGS: Next-generation sequencing
ACE2: Angiotensin converting enzyme 2
CT value: Cycle threshold level
CI: Confidence interval
SGTF: S gene target failure
GMT: Geometric Mean Titre
